# Role of the potassium channels in vasorelaxant effect of asafoetida essential oil

**Published:** 2020

**Authors:** Hassan Esmaeili, Mansour Esmailidehaj, Somayeh Entezari Zarch, Hossein Azizian

**Affiliations:** 1 *Department of Heart, School of Medicine, Gorgan University of Medical Sciences, Gorgan, Iran*; 2 *Department of Physiology, School of Medicine, Shahid Sadoughi University of Medical Sciences, Yazd, Iran*; 3 *Department of Pharmacology, School of Pharmacy, Shahid Sadoughi University of Medical Sciences, Yazd, Iran*

**Keywords:** Asafoetida essential oil, Potassium channels, Aorta, Rats

## Abstract

**Objective:**

In a previous work, we showed that asafoetida essential oil (AEO), from oleo-gum resin of *Ferula asafoetida *L. from the Apiaceae family, has a vasodilatory effect. This effect was both endothelium-dependent and endothelium-independent. The present study was designed to determine whether potassium channels and intracellular calcium release contribute to AEO-induced vasodilation.

**Materials and Methods:**

Rats' thoracic aorta were isolated and denuded. Following induction of contraction by potassium chloride (60 mM), concentration-response curve was plotted by the cumulative addition of AEO (0.625-80 µl/l to the medium of rings. The vasodilatory effect of AEO was assessed before and after addition of phenylephrine and potassium channel blockers (including barium chloride (BC), 4-aminopyridine (4A) and glibenclamide (Gl)).

**Results:**

AEO relaxed the precontracted rings in a concentration-dependent manner (IC50=23 µl/l). All potassium channel blockers significantly attenuated the vasodilatory activity of AEO when they were added to rings medium before addition of KCl (p<0.01, 4A and Gl groups and p< 0.001, BC group vs. control group) but not after that. In contrast to K channel blockers, adding AEO before or after phenylephrine, the tension was reduced significantly (p<0.05 vs. the control group).

**Conclusion:**

The findings of this study indicated that the vasodilatory effect of AEO on denuded-endothelium aortic ring was mediated through activation of potassium channels and reduced intracellular calcium release.

## Introduction

Considering the high-cost of conventional medical therapies and preference of most people to traditional medicine, folk medicine has gained public interest as an alternative therapy in primary health care systems (Mala et al., 2018 ▶)* Ferula asafoetida* is a herbaceous perennial herb of the Apiaceae family with an unpleasant smell. This plant, which is mainly native to Iran and Afghanistan, has mass or carrot-like roots and grows to about 2 meters (Iranshahy and Iranshahi, 2011 ▶; Mahendra and Bisht, 2012 ▶; Amalraj and Gopi, 2017 ▶). Before flowering, the roots and stems of the plant are excised in the summer. A milky-like exudate that drops out from the location of the excision, is called asafoetida (Iranshahy and Iranshahi, 2011 ▶; Mahendra and Bisht, 2012 ▶; Amalraj and Gopi, 2017 ▶). It is called "*Anghuze*" in Iran (Iranshahy and Iranshahi, 2011 ▶). There are two forms of asafoetida: tear and mass. The most common form in the market is mass . Asafoetida has three main fractions:1) resin fraction (40-60%) mainly contains ferulic acids and its esters, coumarins and other terpenoids; 2) gum fraction (25%) mainly contains glucose, galactose, L-arabinose, glucuronic acid, polysaccharides and glycoproteins; and 3) volatile oil fraction (10-17%) mainly contains disulfide compounds, monoterpenes and other volatile terpenoids (Fatehi, Farifteh, and Fatehi-Hassanabad, 2004 ▶; Iranshahy and Iranshahi, 2011 ▶). Sulfur-containing compounds account for its smell (El Deeb, et al. 2012 ▶).

In traditional medicine, asafoetida is used to treat whooping cough, menstrual disorders, asthma, heart disease, intestinal parasites and influenza in different countries (Iranshahy and Iranshahi, 2011 ▶; Ross, 2003 ▶; Eigner and Scholz, 1999 ▶). Experimental studies showed that asafoetida and its preparations have antioxidant (Vijayalakshmi et al., 2012 ▶; Saleem, Alam, and Sultana, 2001 ▶), neuroprotective (Homayouni Moghadam et al., 2014), antimicrobial (El Deeb, et al., 2012 ▶; Divya et al., 2014 ▶), anticancer (Saleem, Alam, and Sultana, 2001 ▶; Bagheri et al., 2017 ▶), antispasmodic (Fatehi, Farifteh, and Fatehi-Hassanabad, 2004 ▶; Bagheri et al. 2014 ▶), and smooth muscle relaxing effects (Bayrami et al., 2013 ▶; Kiyanmehr et al., 2016 ▶).

Potency of asafetida in smooth muscle relaxation was established by previous studies. Some researchers showed that asafoetida and its seeds essential oil had a relaxing effect on ileum smooth muscle of rats and guinea pigs (Fatehi, Farifteh, and Fatehi-Hassanabad, 2004 ▶; Bagheri et al.2014 ▶) and trachea smooth muscle of guinea pigs (Bayrami et al., 2013 ▶; Kiyanmehr et al., 2016 ▶). In 2004, Fatehi et al reported that intravenous administration of aqueous extract of asafoetida into anesthetic rats had hypotensive effects (Fatehi, Farifteh, and Fatehi-Hassanabad, 2004 ▶). Recently, we observed that asafoetida essential oil has a relaxatory effect on the smooth muscle of rat thoracic aorta. Since, the vasodilatory effect of asafetida essential oil was weakened in the presence of nitric oxide synthase and cyclooxygenase inhibitor, it was suggested that the effect is both endothelium-dependent and endothelium-independent (Esmaeili et al., 2017 ▶). 

Considering the above information and the increased use of herbal medicine to treat hypertension, in the present study, we investigated the role of smooth muscle membrane potassium and calcium channels as well as intracellular calcium release in vasodilatory effect of AEO.

## Materials and Methods

**Reagents**

Barium chloride, 4-aminopyridine, glibenclamide, acetylcholine and phenylephrine were purchased from Sigma Chemical Co (USA). All other reagents were of analytical grade.

**Animals**

Adult male Wistar rats (weight range 250-300 g), provided by animal house of Shahid Saduoghi University of Medical Sciences, Yazd, Iran, were used. They were kept under standard conditions (dark and light cycle of 12-12 hr, temperature of 22±2°C and humidity of 55%). All animals had free access to tap water and food. All procedures done in animals were according to the international guidelines for the Care and Use of Laboratory Animals and approved by the Ethic Committee of Shahid Sadoughi University of Medical Sciences, Yazd, Iran (IR.SSU.MEDICINE.REC.1395.217).

**Preparation of AEO**

In order to provide essential oil, asafetida (*Ferula asafoetida* oleo-gum resin) was collected from Dorbid area (Yazd, Iran) at the end of spring 2017. The specimen was identified by Botany department, Faculty of Pharmacy, Shahid Sadoughi University of Medical Sciences, Yazd, Iran. A voucher specimen was kept in (A2343) at the Herbarium of the Herbal Medicine Research Center of Shahid Sadoughi University of Medical Sciences, Yazd, Iran. Afterward, 100 g of sample was soaked in distilled water for 24 hr and then extracted by a Clevenger apparatus. The essential oil was extracted using sodium sulfate, and finally stored in a dark container at 4°C until used.

**Preparation of isolated rat thoracic aorta**

Animals were anesthetized by sodium pentobarbital (50 mg/kg, intraperitoneal). Then, the descending thoracic aorta was removed and placed in normal Krebs physiological solution. Thereafter, the aorta was cleaned from adhering adipose and connective tissues and cut into 3-4 mm rings. The rings were mounted between two stainless steel hooks in 50-ml organ bath (Bio-systems, UK) containing Krebs solution with the composition of (mM) NaCl (118), KCl (4.7), CaCl_2_ (1.6), MgSO_4_ (1.2), KH_2_PO_4_ (1.2), NaHCO_3_ (25) and glucose (11). Krebs solution was maintained at 37^o^C at pH 7.4 and continuously bubbled with carbogen (95% O_2_, 5% CO_2_). Using a force-displacement transducer myograph (F-60, Biosystems, UK) connected to a PowerLab data acquisition system (PowerLab-26T, ADInstruments, Australia), isometric contraction of aortic rings was recorded (lab chart pro 7 software). A tension of 1 g was applied to rings and maintained for 60 min to reach a steady state. During this period, the incubation medium was replaced every 20 min. Before each experiment, the endothelium (i.e. internal layer of the vessel) was mechanically removed by rubbing a cotton thread inside the rings. The absence of endothelium was verified by the absence of the relaxing effect of acetylcholine (10 µm) on rings precontracted by phenylephrine (0.1 µm). Finally, the denuded rings were washed twice with Krebs physiological solution so that it was relaxed completely.

In order to determine the involvement of calcium influx and calcium mobilization from intracellular sources in the vasorelaxant effect of AEO, Ca-free medium was used. It was prepared by replacing CaCl_2_ in normal Krebs with an equimolar concentration of MgCl_2_. EDTA (0.02 mM) was added to the medium to chelate any other free calcium. 

**Experimental groups**

In order to complete this study, six sets of experiments were performed as follow:

1. Determination of IC50 of AEO (set 1). After reaching the steady state by using a tension of 1 g, the aortic rings were precontracted with high KCl (60 mM), and the relaxant responses to AEO at different concentrations (1.25 to 80 µl) were recorded by adding cumulative doses of AEO to the medium of organ bath at 15 min intervals between successive doses. Dose-response curves were plotted as a percent of contractile response to KCl against logarithmic concentrations of AEO (n=6 for each group).

2. Determination of the role of potassium channels in vasodilatory effect of AEO (sets 2 and 3). After reaching the steady state induced by the tension of 1 g, the aortic rings were precontracted with high KCl (60 mM), and then exposed to a single dose of AEO (23 µl). Potassium channel blockers were added into the medium 20 min before (set 2) and after (set 3) adding KCl (n=6 for each group).

3*. *Determination of the role of calcium channels in vasodilatory effect of AEO (set 4). After reaching the steady state induced by the tension of 1 g, in the presence of calcium-free medium, the aortic rings were precontracted with high KCl (60 mM), and then exposed to a single dose of AEO (23 µl) or vehicle as a solvent. After 20 min, calcium chloride was added to the medium (n=6 for each group).

4. Determination of the effect of AEO on calcium release from intracellular sources (sets 5 and 6). After reaching the steady state induced by the tension of 1 g, in the presence of calcium-free medium, phenylephrine (1 µM) was added to the medium. AEO was added to the medium 20 min before (set 5) and after (set 6) adding phenylephrine. 

**Statistical analysis**

The data was analyzed using GraphPad Prism version 7 for Windows (GraphPad Software, La Jolla California USA). The data is displayed as mean±SEM. To determine the IC_50_ of AEO, the data was transformed to the logarithm dose and then IC_50_ was determined using nonlinear regression. To analyze the effect of AEO on the activity of potassium channels, influx of calcium and calcium mobilization from intracellular resources, statistical analysis was performed using student *t* test. A p value less than 0.05 was considered statistically significant.

## Results

**Determination of IC**_50_ **of AEO**

**Figure 1:**
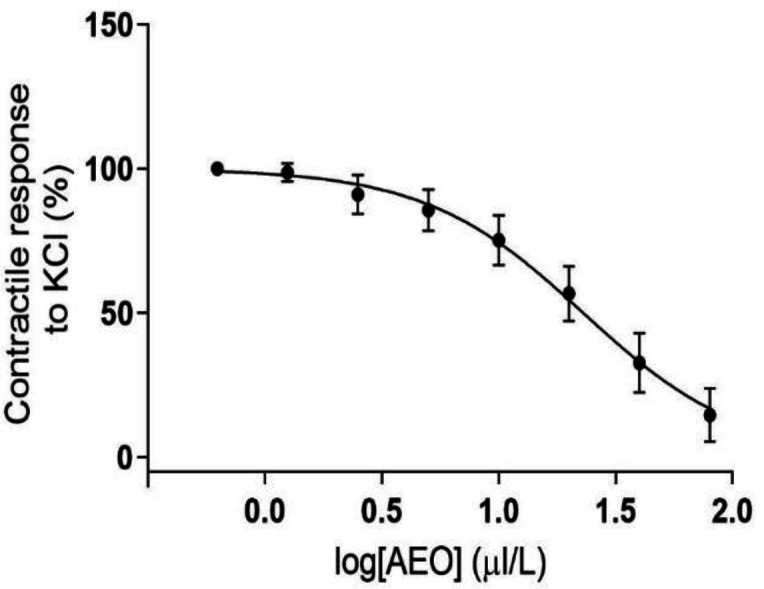
The effect of cumulative concentrations of asafoetida essential oil (AEO) on the contractile response of rat thoracic aorta to KCl (60 mM) and determination of IC50 of AEO. Data is shown as mean±SEM

Figure 1 shows the effect of addition of cumulative doses of AEO on KCl-induced contractions. After one hr stabilization to the force of 1 g, the rings were subjected to KCl (60 mM) for 20 min. This contraction force was considered 100% tension. Then, cumulative doses of AEO were added in seven 15-min steps. As shown in Figure 1, IC_50_ was 23 μl/l. IC_50_ is the concentration of AEO that reduced the contraction force of precontracted aortic rings by 50%. This dose (23 μl/l) was used forever.

**The effect of inhibition of potassium channels on AEO vasodilatory activity**

**Figure 2:**
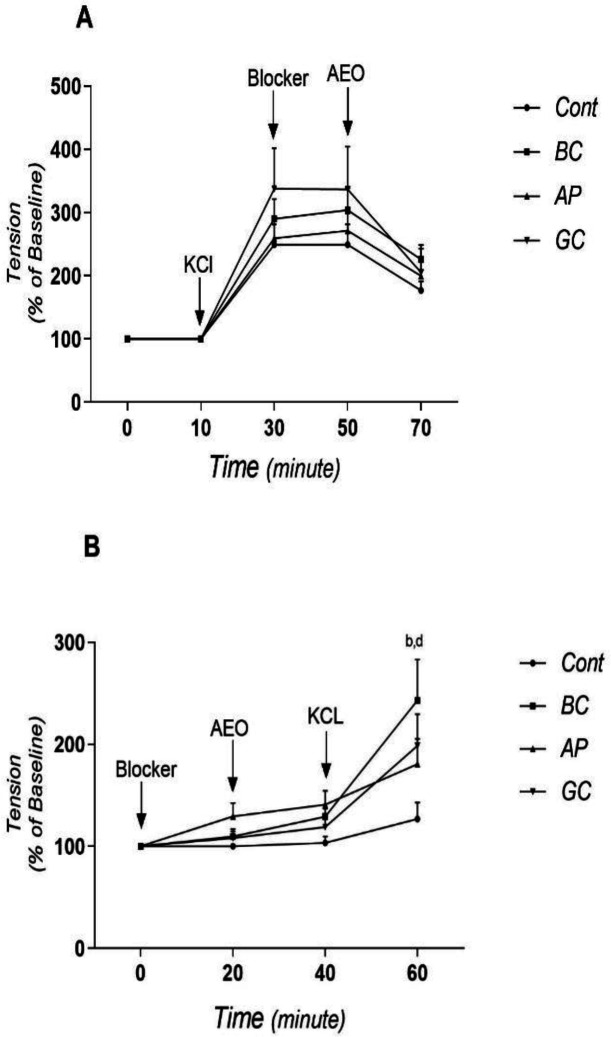
The effect of asafoetida essential oil (AEO) on the activity of transmembrane potassium channels in rat thoracic aorta. A, after contraction induced by KCl (60 mM); B, before contraction induced by KCl (60 mM). Data is shown as mean±SEM. Cont, control group; AP, 4-aminopyridine (blocker of voltage-gated potassium channels); BC, barium chloride (blocker of inward-rectifying potassium channels); GC, glibenclamide (blocker of ATP-sensitive potassium channels). b, p< 0.01 and d, p< 0.001 vs. control group

As Figure 2A shows, KCl significantly increased the tension in rings in all groups, without any significant difference between groups. Adding potassium channel blockers did not have any significant effect on the tension of rings in none of the group. AEO reduced the magnitude of KCl-induced contractions in all groups without any marked difference between groups. 

Figure 2B is similar to Figure 2A but KCl was added to the ring media after adding AEO. Potassium channel blockers and AEO did not have any significant effect on the basal tension in none of the groups. The tension was not increased significantly following KCl addition, but increased markedly in all groups added potassium channel blockers to their medium (p<0.05), especially in BC groups (p<0.01).

**The effect of AEO on the influx of calcium through smooth muscle membrane calcium channels**

**Figure 3:**
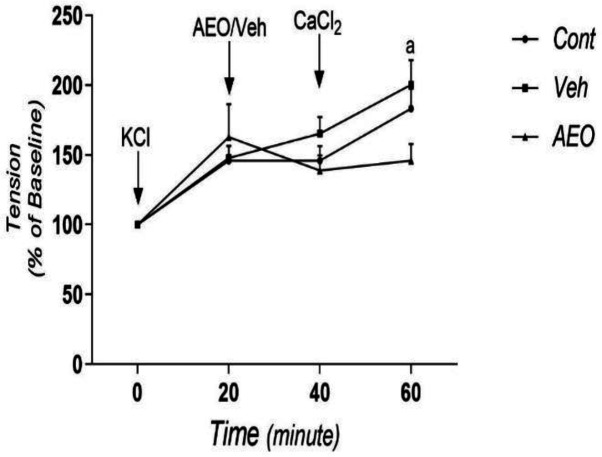
The effect of asafoetida essential oil (AEO) on the influx of calcium thorough plasma membrane calcium channels in rat thoracic aorta. KCl, potassium chloride (60 mM); CaCl_2_, calcium chloride (1.25 mM); cont, control group; Veh, vehicle group; Data is shown as mean±SEM. a, p<0.05 vs. control group

In order to evaluate the effect of AEO on the influx of calcium channels, the steady-state tension of rings in a calcium-free medium was considered 100% i.e. baseline). Then, the rings were contracted by KCl (60 mM) and 20 min later, they were exposed to AEO for 20 min. Figure 3 indicates that AEO had no considerable effect on KCl-induced force in the absence of calcium. Replacing the calcium chloride of medium (1.25 mM), significantly increased the force of contraction in the control and vehicle (as a solvent) groups (p<0.05), but not in AEO group (Figure 3).

**The effect of AEO on the intracellular calcium release**

To determine the effect of AEO on the release of calcium from intracellular resources, the rings were first subjected to calcium-free medium and next to phenylephrine (1 µM). 

**Figure 4:**
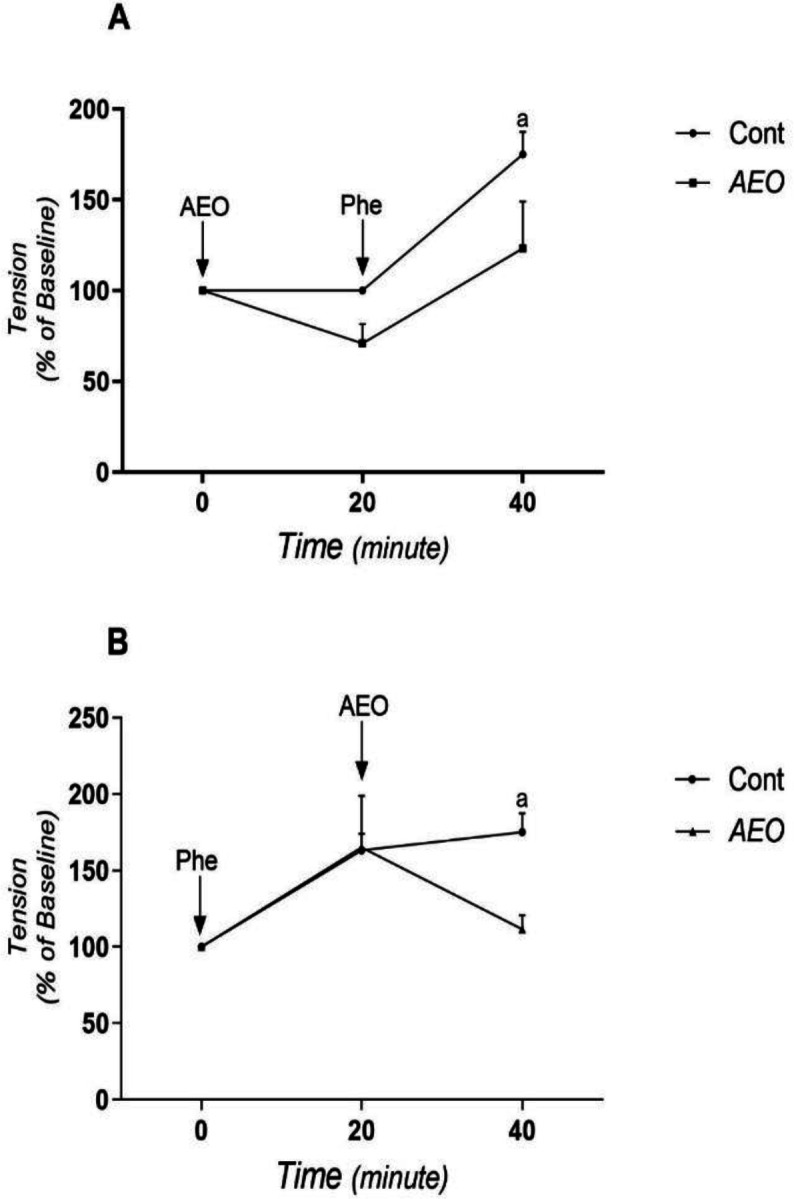
The effect of asafoetida essential oil (AEO) on the release of calcium from sarcoplasmic reticulum in rat thoracic aorta. A, after contraction induced by phenylephrine; B, before contraction induced by phenylephrine. Cont, control group; Phe, phenylephrine; Base, baseline tension of aortic rings to the force of 1 g that considered as tension of 100%. Data is shown as mean±SEM. a, p< 0.01 vs. control group

In Figure 4A, the tension of rings at the steady-state in calcium-free medium was considered 100% (baseline). Then, the rings were exposed to AEO. AEO reduced the ring tension without a significant difference compared to the control group. Next, the rings were exposed to phenylephrine in the presence of AEO. AEO reduced the magnitude of phenylephrine-induced contractions in comparison to the control group (p<0.05).

Figure 4B is similar to the Figure 4A but the rings were first exposed to phenylephrine and then to AEO. As Figure 4B shows, phenylephrine significantly increased the force of rings. Compared to the control group, AEO significantly reduced the magnitude of phenylephrine-induced contractions (p<0.05).

## Discussion

The present study showed that the vasodilatory effect of AEO is mediated through activation of smooth muscle membrane potassium channels and inhibition of calcium channels, as well as inhibiting calcium release from intracellular resources.

Plants are the rich source of substances that affect the biological activities (Fatehi, Farifteh, and Fatehi-Hassanabad, 2004 ▶; Zahoor et al., 2015 ▶). Some of the available drugs in the market have plant origin (Veeresham, 2012 ▶). It was documented that herbal remedies are much less harmful than synthetic drugs (Pal et al. 2016 ▶). Moreover, people has great interest in the use of herbs and their preparations (Fatehi, Farifteh, and Fatehi-Hassanabad, 2004 ▶). Therefore, research about the effect of herbal preparations, including extracts, essential oils and their individual compounds on biological activities, would be of great importance.

Oleo-gum resin (asafoetida) which is mainly obtained from the root and stem of Ferula asafoetida (Apiaceae family) has three main parts:1) gum (25%), 2) resin (40-64%) and volatile oil (3-17%) (Botsoglou et al., 2010 ▶). Experimental studies showed that asafoetida has some biological activities, including neuroprotective (**Homayouni Moghadam et al., 2014**), spermatogenesis (Bagheri et al., 2015 ▶), memory enhancing potential (Vijayalakshmi et al., 2012 ▶), analgesic (Bagheri, Dashti, and Morshedi, 2014 ▶), and smooth muscle relaxating properties (Bayrami et al., 2013 ▶; Kiyanmehr et al., 2016 ▶).

In a few studies, the effects of asafoetida and its essential oil on the relaxation of smooth muscles of trachea and ileum of guinea pigs and rats were reported (Khazdair and Boskabady, 2015 ▶). In 2011, Gholamnezhad and co-workers indicated that aqueous extract of asafoetida at the doses of 2.5, 5 and 10 mg/ml attenuated the maximum contraction response of guinea pig's trachea to metacholine (Gholamnezhad et al., 2011 ▶). They stated that such effects are mediated through blocking muscarinic receptors (Gholamnezhad et al., 2011 ▶). Bayrami et al. in 2013, reported that aqueous extract of asafoetida at the doses of 2.5, 5 and 10 mg/ml reduced the magnitude of contractions caused by potassium chloride and methacholine in guinea pig's trachea (Bayrami et al. 2013 ▶). These effects were comparable to those of theophylline as a standard bronchodilator (Bayrami et al., 2013 ▶). Fatehi and colleagues reported that intravenous administration of asafoetida extract had hypotensive effect in anesthetized rats (Fatehi, Farifteh, and Fatehi-Hassanabad, 2004 ▶).

**Figure 5:**
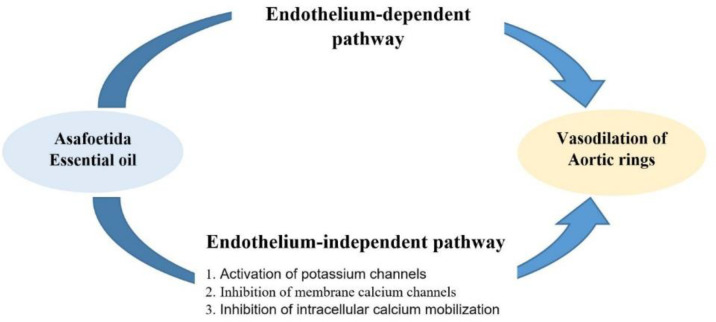
A scheme of the mechanisms involved in asafoetida essential oil’s vasodilatory effect

 This effect seems to be mediated through the relaxation of vascular smooth muscle (Fatehi, Farifteh, and Fatehi-Hassanabad, 2004 ▶). In our laboratory, intravenous injection of asafoetida essential oil, at lower doses, did not have any effect on the arterial blood pressure of anesthetized rats, but it rapidly recovered them from anesthesia at higher doses (data not published). Bagheri et al. in 2014, reported that the essential oil of asafoetida seed, at the concentration of 0.2 and 0.3%, significantly reduced acetylcholine-induced ileum contractions in rats (Bagheri et al., 2014 ▶). Recently, we reported that AEO has vasodilatory effects on isolated rat thoracic aorta. As the inhibition of endothelial nitric oxide synthase by L-NAME and cyclooxygenase by indomethacin significantly, but not completely, attenuated the vasodilatory effect of AEO, these effects were suggested to be mediated thorough endothelium-dependent and endothelium-independent pathways. In the present study, the IC_50_ of AEO was 23 µl/l (0.0023%) that was 100-fold less than that shown in Bagheri’s group work (Bagheri et al., 2014 ▶). This difference may be related to the part of the plant used. 

Several mechanisms involved in the relaxation of vascular smooth muscles including the release of endothelin, nitric oxide, prostaglandins and endothelium-derived vascular relaxing factor, inhibition of calcium channels, and activation of potassium channels (Lee et al., 2015 ▶; Peixoto et al., 2017 ▶). In a previous study, we reported that the AEO has a direct (on smooth muscle) and an indirect (release of nitric oxide and prostacycline) effect on the vascular smooth muscle (Esmaeili et al., 2017 ▶). In the current study, the effect of AEO on the activity of potassium channels was investigated. Closure of potassium channels would lead to vascular contraction thorough depolarizing the plasma membrane and then activation of the calcium channels. Potassium channels in the plasma membrane of smooth muscle cells are divided into four main categories: 1) voltage-gated potassium channels which are blocked by 4-aminopyridine, 2) inward-rectifying potassium channels which are blocked by barium chloride, 3) ATP-sensitive potassium channels which are blocked by glibenclamide, and 4) calcium-activated potassium channels which are blocked by tetraethylammonium (Xue et al., 2011 ▶; Silva et al., 2015 ▶; Sobey, 2001 ▶). In this work, two methods were used to evaluate the role of potassium channels in vasodilatory effect of AEO:1) Blocking the potassium channels before exposing the pre-contracted aortic rings to AEO, and 2) blocking the potassium channels before exposing the rings to AEO and KCl. When the aortic rings were exposed to blockers and AEO respectively, and then contracted by KCl, the contractile force in the potassium channel blocker groups was significantly higher than the AEO group (Figure 2B). This finding suggests that AEO induced its relaxing effects thorough activation (opening) of potassium channels. But, when the rings were first contracted by KCl and then exposed to the potassium channel blockers and AEO, respectively, the contractile force decreased significantly in all experimental groups and there was no significant difference between the groups that received blockers and the AEO group. We do not have any particular explanation for this work. 

The most important factor contributing to smooth muscle contraction and relaxation is the intracellular calcium concentration. Binding of calcium to calmodulin and increasing the activity of the myosin-light chain kinase cause the binding of myosin head to actin and ultimately contraction. The vasoconstriction decreases the flow of blood vessels by narrowing the diameter of the vessel. Calcium passes through the plasma membrane in two ways and enters the cell: 1) directly across the voltage-dependent calcium channels (VDCC) and 2) indirectly thorough the receptor-operated calcium channels (ROCC) (Dekanski et al., 2011 ▶; Lee et al., 2015 ▶; Amberg and Navedo 2013 ▶). In the current study, it was shown that AEO greatly reduced the influx of calcium into the vascular smooth muscle cells. It is not known what type of the calcium channels is blocked by AEO. Therefore, there is a need to search in this field in the future. It remains to be elucidated which compounds in AEO mediated the vasodilatory effect of AEO. 

In addition to the influx of calcium through the cell membrane, the release of calcium from intracellular resources like sarcoplasmic reticulum (SR) also plays a crucial role in the smooth muscle contraction process. The release of calcium from SR is mediated thorough two types of calcium channels: 1) ryanodine receptors and 2) inositol triphosphate (IP3) receptors (Amberg and Navedo, 2013 ▶). In order to evaluate the effect of AEO on the calcium release from SR, phenylephrine and calcium-free medium was used. Phenylephrine first leads to smooth muscle contraction by releasing calcium ions from intracellular resources and then to sustained tonic contraction by stimulating the ROCC (Silva et al. 2015 ▶; Ajay, Gilani, and Mustafa 2003 ▶). Exposing the aortic rings to AEO, before and after adding phenylephrine to the medium, significantly reduced the magnitude of phenylephrine-induced contractions. In this study, it is not clear how the effective compounds of AEO did weaken the phenylephrine-induced contractions:1) thorough inhibition of ryanodine receptors? or 2) inhibition of IP3 receptors? and/or 3) competition with the binding of phenylephrine to its receptors? To clarify these mechanisms, further research is needed in the future.

Several studies examined the compounds in AEO. The main ingredients were disulfides and to some extent, monotropenes. In our previous study, the analysis of AEO using GC-MS showed that disulfides are the main components of AEO (Esmaeili et al., 2017 ▶). Disulfides seem to be responsible for the biological effects of AEO. 

According to our previous (Esmaeili et al., 2017 ▶) and current works, we present a scheme like Figure 5 to display the proposal mechanisms involved in AEO-induced vasodilation.

In conclusion, the main findings of the present study indicated that AEO has a potent vasodilatory effect on rat's thoracic aorta. The endothelium-independent vasodilatory effects of AEO are mediated through activation of potassium channels, inhibition of plasma membrane calcium channels and inhibition of calcium release from intracellular sources of vascular smooth muscles. Therefore, it is necessary to find the effective compounds of AEO in the future that might be effective in management of hypertension.
